# Professional Identity and Career Adaptability among Chinese Engineering Students: The Mediating Role of Learning Engagement

**DOI:** 10.3390/bs13060480

**Published:** 2023-06-07

**Authors:** Xinqiao Liu, Xinyu Ji, Yifan Zhang, Wenjuan Gao

**Affiliations:** 1 School of Education, Tianjin University, Tianjin 300350, China; 2 Institute of Higher Education, Beihang University, Beijing 100191, China; 3 School of Public Administration, Beihang University, Beijing 100191, China; 4 Research Centre for Beijing Higher Education Development, Beihang University, Beijing 100191, China

**Keywords:** professional identity, career adaptability, learning engagement, college students, mediation effect model

## Abstract

Due to the rapid development of science and technology, economic development has changed dramatically, resulting in the transformation of career characteristics. Individuals need to convey a higher career adaptability than ever before in order to face the rapid changes brought by development. Especially for college students in the critical period of career development, having good career adaptability is of great significance to their future career choice and development. This study conducted a cross-sectional survey of 692 engineering undergraduates at a top engineering university in China and used the data to investigate the relationship between the professional identity (professional interest, professional strength, career prospects, and professional satisfaction) and career adaptability of college students, as well as to discuss the mediating role of learning engagement in the relationship between professional identity and career adaptability. The results of the correlation analysis showed that professional identity was positively correlated with career adaptability. The mediation effect model indicated that learning engagement played a mediating role in the relationship between the professional identity and career adaptability of Chinese college students. In other words, professional identity had a direct positive impact on career adaptability, while professional identity, mediated by learning engagement, had a positive impact on career adaptability. The study recommends that colleges provide students with a more conducive academic environment and more opportunities for career practice. We also encourage educators to provide more emotional support and identity for students to enhance students’ career adaptability by creating a favorable academic and emotional atmosphere.

## 1. Introduction

With the rapid development of society, the future development of individuals is faced with various unpredictable changes, and it is necessary to improve career adaptability to promote better development in a changing career path. Career adaptability is a core competence of individuals to achieve career success [1]. This concept originated from the revision of vocational maturity theory [2]. In 1981, Super and Knasel further stated that career adaptability refers to an individual’s ability to cope with changes in occupational roles and to balance stress in the work environment [3]. Super et al. [4] noted that career adaptability was the ability to respond appropriately to uncertain reality, and that the interaction between individuals and the environment should be highlighted. Since then, the concept of career adaptability has gradually replaced vocational maturity. Savickas subsequently expanded the concept of career adaptability [5,6,7] and defined it as an individual’s ability to prepare and cope with predictable career tasks, career roles, and career changes or unpredictable career problems. In 2005, Savickas constructed a career adaptability model from four dimensions: concern, control, curiosity, and confidence, and each dimension contained a question [7]: Do I have a future? Who owns my future? What do I want to do in the future? Can I do it?

In recent years, academia has paid much attention to the construction of career adaptability and its influence mechanism, but most studies have focused on the predictive effect of career adaptability on outcomes. For instance, from the perspective of concept and experience, Hirschi et al. [8] explored the relationship between different operating modes of career adaptability in terms of attention, control, curiosity, and confidence. Other scholars have explored the prediction of career adaptability on outcome variables such as career choice [9], life satisfaction [10], personal growth, and well-being [11].

College is a critical period for the development and transformation of career roles [12]. College students need to adjust themselves so that they can smoothly transition from students to professionals and gradually meet the requirements of their careers and complete career activities. Career adaptability is a significant ability to help college students develop and plays a role in college students’ career construction and decision-making [13,14]. Therefore, career adaptability is a critical variable for observing the lifelong career development of college students [1]. Engineering students account for 34.02% of Chinese college students, which represents a significant group for national development, and good career adaptability is particularly critical for their employment. Most studies take all students as samples, but there are few studies on this large group. Therefore, this study aims to know more about the career adaptability of engineering students in China.

Career adaptability in college is affected by various factors in study and life [15,16,17]. In China, when choosing college majors after high school graduation, students are usually restricted by the enrollment plan of colleges and disturbed by the ranking of colleges and the popularity of majors. Under such complicated conditions, many students end up choosing majors that they do not like. Low professional identity directly leads to a decrease in their learning engagement during college, which may affect the development of their career adaptability. Based on this, the present study focuses on college students’ professional identity and explores the influence mechanism of professional identity and learning engagement on career adaptability.

### 1.1. The Relationship between Professional Identity and Career Adaptability

Salling Olesen [18] defined professional identity as the balance and consistency that an individual could feel toward his or her major in real life. Professional identity is a significant tool to judge an individual’s career development [19], which affects his or her future career choice and attitude toward the careers to be engaged. Welmond’s [20] study revealed that students could combine the professional knowledge they had learned with their interest, had a positive desire to explore their future career, and expected to make contributions to their professional field. In the existing research, we noticed some relevant evidence. For instance, Šverko and Babarović [21] applied the Personal Globe Inventory Scale, the HEXSCO-60 Scale, and the Career Adapt-Abilities Scale (CAAS) to a sample of 981 high school graduates and discussed the relationship between interest and career adaptability. The results suggested that career adaptability and general interests had a weak positive correlation. People who showed higher interest in prestigious careers tended to have higher career adaptability, and were more curious about future career options, more confident in pursuing professional tasks, more concerned about their careers, and more willing to prepare for their future careers.

Ledyandini et al. [22] used quantitative methods in a study of students majoring in accounting at Gorontalo Provincial College, aiming to determine the effect of gender, financial reward, and professional identity on career choice, and they discovered that the simultaneous variables of gender, financial reward, and professional identity had a significant positive effect on the choice of accounting career. Leung et al. [23] conducted a two-stage test for college students in Hong Kong. They completed adaptive readiness (interest and competence flexibility) and adaptability resource (Career Adapt-Ability Scale, CAAS) measures in their sophomore or junior year, respectively. The adapting responses (life-skills competencies) and adaptation result (presence of life purposes, career decision-making difficulties) measures were conducted at the end of the senior year. The results showed that interest could prompt the stimulation of adaptive resources and that adaptive resources could directly or indirectly promote adaptive responses. From the perspective of reinforcement sensitivity theory (RST), Corr and Mutinelli [24] explored the relationship between the related factors of career (career adaptability, career optimism, and perceived knowledge) and three main systems of the reinforcement sensitivity theory of personality (Behavioral Approach System, Fight-Flight-Freeze System, and Behavioral Inhibition System). The results indicated that three related factors of career were positively correlated with Behavioral Approach System scores, mainly with reward interest. Burnik and Košir [25] introduced a novel industrial design program that provided students with industrial experience in the study. The students who participated in the program showed a higher professional responsibility than the average level, which indicated that understanding career prospects and building professional identity had a positive effect on students’ future career adaptation and choice. Urbanaviciute et al. [26] discussed the relationship between occupational barriers and vocational identity commitment based on a sample of Lithuanian undergraduates and explored the mediating role of academic major satisfaction. The results revealed that perceived occupational barriers were negatively correlated with academic major satisfaction, and occupational barriers were negatively correlated with vocational identity commitment through academic major satisfaction. In other words, when the students had higher academic major satisfaction, they would have lower perceived difficulty in achieving career goals and better adapt to their future career.

In the present study, we discuss the relationship between professional identity and career adaptability. Professional identity is a variable composed of professional interest, professional strength, career prospects, and professional satisfaction. According to the review of the literature, we notice that although there are some relevant empirical studies, there is still a lack of direct research on the relationship between the professional identity and career adaptability of engineering college students.

### 1.2. The Mediating Role of Learning Engagement

In a few previous studies, we noticed some empirical evidence that learning engagement is related to occupational adaptability and professional identity. For instance, Rossier et al. [27] conducted a study on Swiss individuals and discovered that career adaptability resources and work outcomes (e.g., career involvement) had a positive correlation. Tladinyane and Merwe [28] also discovered a significant relationship between career adaptability and career engagement. Reinforcement theory proposes that continuous stimulation leads to changes in cognitive and behavioral responses in the brain and increases the possibility of such behavior. Investing time in professional learning is an effective means of consolidating students’ professional development. Learning in class and self-study after class can both consolidate the professional knowledge that they have acquired, thus playing a positive role in career adaptability [29]. Kumar and Choudhury [30] noticed that the time that students spent on homework each day positively affected their learning ability. Xiuyun [31] revealed the influence mechanism of professional cognition on decision-making difficulties in the career. These studies suggest that higher professional identity leads to lower difficulty in making decisions. In other words, when college students learn more about their majors, they have a higher professional identity. A high professional identity makes college students more enthusiastic about learning and more committed to learning, and gives them more professional advantages than other students in future career selection. Based on the job demands–resources model, Gupta [32] investigated the mediating effect of career engagement on the perceived career support and career performance of young knowledge workers as well as the mediating effect on career adaptability and career performance. The results showed that career engagement, career adaptability, and career performance were significantly positively correlated. However, Cotter and Fouad [33] did not find a significant correlation between career adaptability and career engagement in their research on layoff survivors.

There are some definitions of learning engagement. The study uses Kuh’s [34] definition that “the time and energy students devote to educationally sound activities inside and outside of the classroom, and the policies and practices that institutions use to induce students to take part in these activities”. Under this definition, we use “study in class” and “study after class” as components of learning engagement. Although previous studies helped us find a positive correlation between learning engagement, professional identity, and career adaptability, few studies focused on the mediating effect of learning engagement on professional identity and career adaptability and more efforts are still needed, which can prompt school administrators to better serve the needs of student growth and development.

### 1.3. Research Design

In previous studies, we notice that the sub-factors that constitute professional identity (professional interest, professional strength, career prospects, and professional satisfaction) are positively correlated with career adaptability. These results arouse our interest in the direct relationship between professional identity and career adaptability. Therefore, we propose the following hypotheses:

**Hypothesis** **1.** *Professional identity and career adaptability are significantly positively correlated, and the improvement of professional identity significantly leads to the improvement of career adaptability (professional identity → career adaptability).*

Since existing studies have paid little attention to the role of learning engagement in the relationship between professional identity and career adaptability, we further explore the mediating role of learning engagement and clarify the relationship between professional identity and the career adaptability of engineering students, as well as the mediating role of learning engagement. We propose the following research hypothesis:

**Hypothesis** **2.** *The improvement of learning engagement significantly promotes the improvement of career adaptability (learning engagement → career adaptability), and learning engagement plays a mediating role between professional identity and career adaptability (professional identity → learning engagement → career adaptability).*

**Figure 1:**
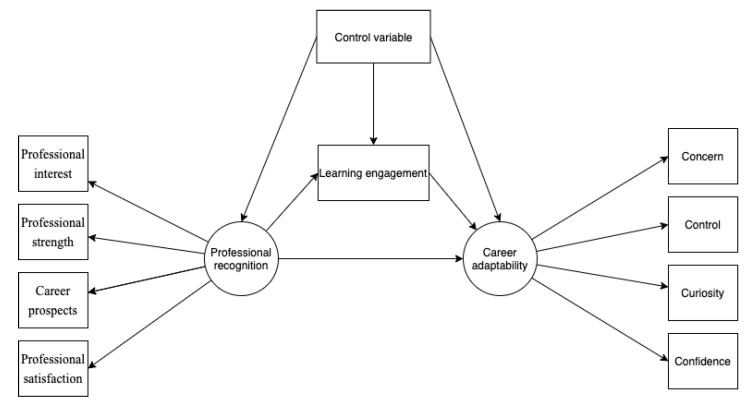
Theoretical research framework.

Previous studies have discovered that career adaptability is correlated with gender. Most studies indicated that gender differences exist in career adaptability [35,36], while a few studies stated no significant gender differences [1]. Previous studies have also shown the possibility of differences between ages [24,37]. In this study, these two variables are set as control variables in the model construction. According to the relevant theories and literature, a theoretical framework is formed (Figure 1).

## 2. Methods

### 2.1. Participants

The study used data from an online survey of engineering undergraduates in China. The survey adopted a stratified random sampling method. Five engineering schools were randomly selected from 25 engineering schools of the university. The participants were randomly selected according to the size of schools and the number of students in different academic years, as well as stratified by average education level and population proportion. The questionnaire had six parts, including students’ basic information, experience before college, course learning, college satisfaction, physical and mental health, and employment after graduation, aiming to understand the quality of engineering education in China and to provide relevant data support and policy suggestions for the reform and development of China’s emerging engineering education. The study selected a university participating in the survey as a sample and included 692 samples for analysis. The average age of students who participated in the survey was 20.8. There were 153 females and 539 males, accounting for 22.11% and 77.89%, respectively. Seventy-six students were in their first year, accounting for 11.03%. Two hundred and forty students were in their second year, accounting for 34.83%. Two hundred and thirty-one students were in their third year, accounting for 33.53%. One hundred and forty-two students were in the fourth year, accounting for 20.61%. Six hundred and eight students were of Han nationality, accounting for 87.9% of the sample, while 84 students were ethnic minorities, accounting for 12.1%. There were 439 students without siblings and 253 students with siblings, accounting for 63.4% and 36.6%, respectively. One hundred and ten students came from rural areas and 582 students from urban areas, accounting for 15.9% and 84.1%, respectively.

### 2.2. Measures

Professional identity. In the study, three items including “strong professional interest”, “high professional strength”, and “good career prospects”, and 10 items of “high professional satisfaction” were used to constitute the latent variables of professional identity. Each item was evaluated on a seven-point scale, with 1 representing “strongly disagree” and 7 representing “strongly agree”. The average score of these items was calculated to measure students’ professional identity. When the average score was higher, the professional identity was higher. The reliability coefficient α of the professional identity scale was 0.921.

Learning engagement. The study used learning time to measure students’ learning engagement. The questionnaire asked students about their average weekly learning time, including study in class (experimental courses and practice courses) and study after class (homework, reading, etc.) [38,39,40]. By adding students’ study in class and after class, a continuous variable ranging from 0 to 66 was obtained as a proxy variable for learning engagement.

Career adaptability. The items in the career adaptability scale were from the scale compiled by Sibunruang et al. [41], including four subscales of attention, control, curiosity, and confidence and three items for each subscale. Attention was measured using items such as “I will think about what my future looks like”, control was measured using items such as “I can make my own decisions”, curiosity was measured using items such as “I will seek opportunities for personal growth”, and confidence was measured using items such as “I will do things carefully”. A 7-point Likert scale was used, with 1 representing “disagree” and 7 representing “very agree”. The average score was calculated to measure the career adaptability of engineering students, and a higher average score indicated higher career adaptability. The reliability coefficient α of the career adaptability scale was 0.955.

### 2.3. Data Analysis

First, Stata 16.0 was used for descriptive statistical analysis of professional identity, learning engagement (including study in class and study after class), and career adaptability of engineering college students. The mean, standard deviation, minimum, maximum, kurtosis, and skewness of these variables were calculated. Second, we used Stata 16.0 to conduct a correlation analysis on professional identity, learning engagement, career adaptability, and other variables. Finally, we constructed the mediation model in Mplus 8.3 and empirically examined the mediating effect of learning engagement on the relationship between professional identity and career adaptability.

## 3. Results

### 3.1. Descriptive Statistics

Table 1 shows the mean, standard deviation, minimum, maximum, skewness, and kurtosis of career adaptability, learning engagement, and professional identity. Descriptive statistics reveal that the average score of self-reported career adaptability is 61.909, which is higher than the medium level, indicating that students generally think they have relatively good career adaptability. The average learning engagement is 36.142, of which the average weekly study in class is 18.934 h, and the average study after class is 17.208 h. The average score of professional identity was 70.595. Specifically, the average score for engineering students’ professional interest is 4.710, the average score for professional strength is 5.354, the average score for career prospects is 4.585, and the average score for professional satisfaction is 55.947. The results show that the mean values of professional identity, professional interest, professional strength, career prospects, and professional satisfaction, which constitute the variables of professional identity, are all higher than the medium level. Students are satisfied with their majors and recognized for their professional strength. Although the students are interested in the majors, the degree of interest is moderate. According to the rating of career prospects, students are generally not very confident about the career prospects of their major.

### 3.2. Correlation Analysis

Table 2 shows the correlation coefficients of career adaptability, learning engagement, and professional identity (professional interest, professional strength, career prospects, and professional satisfaction). The results indicate that career adaptability is significantly positively correlated with the professional identity of Chinese engineering students (*p* < 0.01). In other words, both professional identity and career adaptability change in the same direction. When professional identity increases by one unit, career adaptability increases by 43.9%. Career adaptability is significantly positively correlated with learning engagement (*p* < 0.01), which means that when learning engagement increases by one unit, career adaptability increases by 21.1%. Career adaptability factors (concern, control, curiosity, confidence) are also significantly positively correlated with learning engagement and professional identity. In addition, professional identity is significantly positively correlated with learning engagement (*p* < 0.01). When professional identity increases, learning engagement will also increase. The above verifies Hypothesis 1. The results suggest that career adaptability is positively correlated with students’ professional interest, professional strength, career prospects, and professional satisfaction (*p* < 0.01). Learning engagement is also positively correlated with students’ professional interest, professional strength, career prospects, and professional satisfaction (*p* < 0.01). Other variables also show a significant positive correlation.

### 3.3. Mediation Model

**Figure 2:**
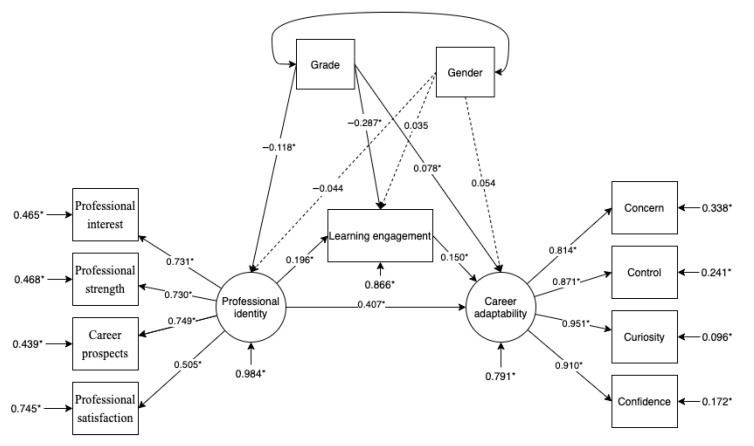
Mediation effect model. Note: * *p* < 0.05.

The study controlled for gender and grade and used a mediation model and bootstrap methods to investigate the mediating role of learning engagement between professional identity and career adaptability. The model fit index was good, with Chi square = 144.245, degree of freedom = 37, RMSEA = 0.065, CFI = 0.970, TLI = 0.956 [42,43]. Table 3 shows the results of the model. In the mediation model, the direct effect of professional identity on career adaptability was 0.407 (*p* < 0.001), the confidence interval was [0.330, 0.544], and the direct effect was significant. The indirect effect was 0.030 (*p* < 0.05), and the confidence interval was [0.013, 0.057], indicating a significant indirect effect. Gender (*p* > 0.1) had no significant impact on career adaptability, while grade (*p* < 0.05) had a significant effect on career adaptability (Figure 2). The above results indicate that learning engagement plays a mediating role in the relationship between career adaptability and the professional identity of Chinese engineering students, which supports Hypothesis 2. Specifically, professional identity has a positive effect on career adaptability through the mediating effect of learning engagement.

## 4. Discussion

This study examined the relationship between the professional identity and career adaptability of Chinese engineering students and identified the mediating role of learning engagement between professional identity and career adaptability. These findings provide new and valuable evidence and supplement the literature from the perspective of engineering education in China.

The results of the descriptive analysis show that the average values of career adaptability, professional identity, and explicit variables (professional interest, professional strength, career prospects, professional interest, professional strength, career prospects, and professional satisfaction) that constitute the latent variables of professional identity are higher than the medium, which suggests that the satisfaction of Chinese engineering students with their majors and career development is generally positive. In the report on learning engagement, the average weekly time spent on study (including study in class and study after class) of Chinese engineering students is 36.142 h. The average time spent on study was 5.163 h every day, and the average time in class was approximately one hour longer than the time after class.

The results of the correlation analysis show a significant positive correlation between professional identity and career adaptation, confirming previous research [22,25]. Specifically, professional interest has a significant positive correlation with career adaptation, which is consistent with previous studies [21,23,24]. According to self-determination theory [44], when students’ self-determination is supported, they will actively learn what they are interested in. This brings some enlightenment to educators: we can explore some new forms of educational practice by promoting the internalization of students’ external motivation and enhancing students’ internal motivation to make students more interested in their major to improve their career adaptability. Professional strength, career prospects, and professional satisfaction are also found to be significantly positively correlated with career adaptability. This is in line with previous studies [25,26].

The study also confirmed the mediating role of learning engagement in the relationship between the professional identity and career adaptability of Chinese engineering students. Based on the mediation effect model, we discovered that professional identity had a direct positive effect on the development of career adaptability after controlling for gender and grade. This finding reaches the same conclusion as previous studies [30,32]. Moreover, it is also found that professional identity can indirectly affect career adaptability through learning engagement, which supports the viewpoint of Rossier, Zecca, Stauffer, Maggiori, and Dauwalder [27]. The reinforcement theory of Skinner [29] seems to explain this result that continuous learning stimulation strengthens students’ professional knowledge and ability, thus achieving the improvement of career adaptability. However, Cotter and Fouad [33] did not find a mediating effect of learning engagement in their study, and we suppose that it might be related to the samples they used in the study that were relatively special (layoff survivors). In addition, unlike previous studies, no gender difference was obtained in this study. Previous studies suggested that gender differences in career adaptability were caused by differences in social support [35,36], and perceived social support promotes the stability of adolescents’ career decisions by providing emotional or tangible support to adolescents [45]. Most studies believed that women generally had better career adaptability, because women were usually more sensitive to their own needs than men, resulting in them feeling more social support [46]. However, no significant effect of gender on career adaptability was discovered in this study, which supports the view of [1] and adds literature to the uncertain gender differences in career adaptability. In this study, the grade has a significant positive impact on career adaptability, which means that older young people have high career adaptability, and this is consistent with previous studies [24,37]. These findings can be explained using the motivational theory of longevity development [47], in which an individual’s primary control capacity (i.e., the ability to align the environment with the self) decreases with age, and an individual’s secondary control capacity (i.e., the desire to align the self with the environment) increases with age to compensate for the decrease in the former.

Based on the above findings, we suggest that universities should provide more courses for students [48] to help students develop human capital and professional identity. Vocational practice should be appropriately increased in future education. Career-oriented activities can increase students’ learning engagement and improve their career awareness and adaptability. At present, some good cases can be learned from. For instance, the Youth@work game introduced by Hummel et al. [49] provides an attractive and inspiring professional game to enable teenagers to obtain vocational learning support, which can improve students’ career adaptability in terms of attention, control, confidence, etc. Immersive professional activities can be a way for students to develop a good sense and thus increase their interest in their major. Skills-based activity is also a good way that companies can provide job opportunities for students, and students can accumulate professional practical experience in advance, which is beneficial to both sides. Moreover, project-based learning has shown positive effects in previous practices. In other words, educational organizers can enhance students’ professional identity and learning engagement through richer teaching content and more effective teaching forms, and improve students’ career adaptability through real assessment and effective guidance.

In addition, based on the affect infusion model [50] and affective events theory [51], the emotions that people experience in the environment can affect their satisfaction. Accordingly, studies have shown that a positive environment [52] and emotional experience [53] are related to students’ professional satisfaction and professional identity. Therefore, we suggest that schools explore a more conducive academic environment for student development, such as student services, library resources, and other aspects that can be further improved. We also suggest that educators (especially supervisors and counselors) establish a strong emotional relationship with students, encourage students, and enhance students’ professional identity by creating a good emotional environment to improve students’ career adaptability. This study can provide enlightenment and reference for future educational practice.

## 5. Limitations

The study has a few limitations. First, the study was carried out based on cross-sectional data. In future studies, we plan to use longitudinal data to examine how variables change over time. Second, the samples in the study are merely from engineering students in Beijing, China, so caution should be exercised when applying these findings to students from other regions or disciplines. Third, the data are from the self-report questionnaire of students, which may lead to some bias in the results.

## 6. Conclusions

First, the study confirms that there is a significant positive correlation between professional identity and career adaptability among Chinese engineering students. Students’ professional interest, professional strength, career prospects, and professional satisfaction are also significantly positively correlated with their career adaptability. Second, the professional identity of Chinese engineering students can positively predict career adaptability, with learning engagement playing a mediating role. It is suggested that colleges pay more attention to subject construction and improve their overall strengths to improve career adaptability among students. Educators of higher education should establish a good emotional connection with students and create a supportive academic and emotional atmosphere to enhance their identity and satisfaction with their majors, enabling students to have higher expectations for future employment. In addition, we recommend that universities explore more diversified methods to help students understand their majors more comprehensively to improve their career adaptability.

## Figures and Tables

**Table 1:** Descriptive statistics of career adaptability, learning engagement, and professional identity.

Variables	N	Mean	Standard Deviation	Min	Max	Skewness	Kurtosis
Career adaptability	692	61.909	12.156	12	84	−0.549	4.370
Learning engagement	692	36.142	14.002	0	66	−0.048	2.815
Study in class	692	18.934	9.480	0	33	−0.255	2.171
Study after class	692	17.208	9.160	0	33	0.264	2.126
Professional identity	692	70.595	12.281	13	91	−0.653	3.716
Professional interest	692	4.710	1.544	1	7	−0.604	3.037
Professional strength	692	5.354	1.461	1	7	−1.049	3.898
Career prospects	692	4.585	1.429	1	7	−0.544	3.161
Professional satisfaction	692	55.947	10.234	10	70	−0.649	3.668

**Table 2:** Correlation analysis of career adaptability, learning engagement, and professional identity.

Variables	1	2	3	4	5	6	7	8	9	10	11
1. Career adaptability	1										
2. Learning engagement	0.211 ***	1									
3. Professional identity	0.439 ***	0.217 ***	1								
4. Attention	0.890 ***	0.203 ***	0.355 ***	1							
5. Control	0.909 ***	0.166 ***	0.421 ***	0.730 ***	1						
6. Curiosity	0.945 ***	0.202 ***	0.412 ***	0.774 ***	0.829 ***	1					
7. Confidence	0.923 ***	0.201 ***	0.428 ***	0.735 ***	0.789 ***	0.870 ***	1				
8. Professional interest	0.283 ***	0.218 ***	0.513 ***	0.244 ***	0.258 ***	0.255 ***	0.282 ***	1			
9. Professional strength	0.262 ***	0.178 ***	0.585 ***	0.195 ***	0.247 ***	0.262 ***	0.263 ***	0.538 ***	1		
10. Career prospects	0.266 ***	0.125 ***	0.556 ***	0.225 ***	0.238 ***	0.261 ***	0.255 ***	0.585 ***	0.553 ***	1	
11. Professional satisfaction	0.410 ***	0.185 ***	0.961 ***	0.330 ***	0.398 ***	0.382 ***	0.398 ***	0.306 ***	0.401 ***	0.360 ***	1

Note: *** *p* < 0.01.

**Table 3:** Mediating effect of learning engagement on the relationship between professional identity and career adaptability among engineering students.

Pathways	Estimate	95%CI		Standard Error	Estimate/Standard Error	*p* Value
		Lower	Upper			
Direct effects						
Professional identity → Career adaptability	0.407	0.330	0.544	0.054	7.557	0.000
Indirect effects						
Professional identity → Learning engagement → Career adaptability	0.030	0.013	0.057	0.010	2.897	0.004

## Data Availability

Data will be made available upon request.
